# Thermophysical Characterization of MgCl_2_·6H_2_O, Xylitol and Erythritol as Phase Change Materials (PCM) for Latent Heat Thermal Energy Storage (LHTES)

**DOI:** 10.3390/ma10040444

**Published:** 2017-04-24

**Authors:** Stephan Höhlein, Andreas König-Haagen, Dieter Brüggemann

**Affiliations:** Chair of Engineering Thermodynamics and Transport Processes (LTTT), Center of Energy Technology (ZET), University of Bayreuth, Universitätsstraße 30, 95440 Bayreuth, Germany; Andreas.Koenig-Haagen@uni-bayreuth.de (A.K.-H.); brueggemann@uni-bayreuth.de (D.B.)

**Keywords:** phase change material, thermal energy storage, latent heat storage, salt hydrate, sugar alcohol, properties, waste heat, DSC, thermal diffusivity, density

## Abstract

The application range of existing real scale mobile thermal storage units with phase change materials (PCM) is restricted by the low phase change temperature of 58 ${}^{\circ }C$ for sodium acetate trihydrate, which is a commonly used storage material. Therefore, only low temperature heat sinks like swimming pools or greenhouses can be supplied. With increasing phase change temperatures, more applications like domestic heating or industrial process heat could be operated. The aim of this study is to find alternative PCM with phase change temperatures between 90 and 150 ${}^{\circ }C$. Temperature dependent thermophysical properties like phase change temperatures and enthalpies, densities and thermal diffusivities are measured for the technical grade purity materials xylitol (C${}_{5}$H${}_{12}$O${}_{5}$), erythritol (C${}_{4}$H${}_{10}$O${}_{4}$) and magnesiumchloride hexahydrate (MCHH, MgCl${}_{2}$·6H${}_{2}$O). The sugar alcohols xylitol and erythritol indicate a large supercooling and different melting regimes. The salt hydrate MgCl${}_{2}$·6H${}_{2}$O seems to be a suitable candidate for practical applications. It has a melting temperature of 115.1 ± 0.1 ${}^{\circ }C$ and a phase change enthalpy of 166.9 ± 1.2 J/g with only 2.8 K supercooling at sample sizes of 100 g. The PCM is stable over 500 repeated melting and solidification cycles at differential scanning calorimeter (DSC) scale with only small changes of the melting enthalpy and temperature.

## 1. Introduction

Phase change materials (PCM) are attractive candidates for storing thermal energy. Due to their high storage density around the phase change temperature, these materials are suitable for applications that require a compact design. One field of application is the transport of waste heat to sites with heat demand. Mobile storage containers benefit from the existing street infrastructure and have the flexibility to react to changes in the heat supply or demand. The existing real scale mobile PCM storage systems, described by Storch and Hauer [1], KTG Group [2] and Deckert et al. [3], commonly use sodium acetate trihydrate as storage material. Novel ideas with other PCM exist but up to now only at numerical and lab scale level or for conception and feasibility studies [4]. The comparatively low melting temperature of about 58 ${}^{\circ }C$ of the sodium acetate trihydrate restricts the utilization of these units to only a few selected low temperature applications like swimming pools or greenhouses. To enhance the range of application, PCM with higher melting points are necessary. Therefore, one aim of this study was to find potential PCM candidates for mobile heat storage applications within a temperature range between 90 and 150 ${}^{\circ }C$. The PCM has to be available at low cost and in large quantities to enable economic utilization in storage applications. Materials of interest are salt hydrates and sugar alcohols, since these materials have comparatively high densities and therefore high volumetric storage densities [5]. As a drawback, supercooling and phase separation can occur. The sugar alcohols, xylitol and erythritol, indicate a melting temperature within the desired temperature range while magnesiumchloride hexahydrate (MCHH) is a potential salt hydrate. At the desired temperature range, there are more potential PCMs available (e.g., (NH${}_{4}$)Al(SO${}_{4}$)·12H${}_{2}$O, Al(NO${}_{3}$)${}_{3}$·9H${}_{2}$O, Mg(NO${}_{3}$)${}_{2}$·6H${}_{2}$O, 33% LiNO${}_{3}$ + 67% KNO${}_{3}$ and some paraffins), but since they are e.g., flammable, toxic or very corrosive, they would be unfavourable for mobile storage containers on the road.

There are several studies concerning the properties of erythritol. Cohen et al. [6] have established a very low hygroscopicity that is very important for the handling of the PCM in ambient conditions. Kaizawa et al. [7] have determined a decomposition temperature of 160 ${}^{\circ }C$, which is the limiting temperature for the use of erythritol as thermal energy storage material. Lopes Jesus et al. [8] have studied the general melting and crystallisation behaviour and reported the occurrence of two different crystalline forms of the solidified erythritol with different melting points. For the supercooling phenomena, Shukla et al. [9] have reported a supercooling of maximum 14 K at sample sizes of 200 g. Sari et al. [10] have measured higher supercooling up to 82 K and Ona et al. [11] up to 54 K with differential scanning calorimeter (DSC) measurements for significant smaller sample sizes of only a few mg. These results reflect the volume dependence of the supercooling phenomena. Bigger volumes contain more potential nucleation sites and therefore a lower supercooling. The same tendency has been observed by Rathgeber et al. [12], who compared DSC and T-history measurements, and Wei and Ohsasa [13]. Cycling experiments to test the thermal stability of erythritol at repeated melting and crystallization has been conducted by Shukla et al. [9] and Agyenim et al. [14]. Both groups have reported a decrease in the latent heat and variations of the phase change temperature during cycling. Measurement of melting and crystallisation enthalpies and temperatures have been performed by several authors and some results are summarized in Table 1. Erythritol is used as storage material for a variety of applications. Agyenim et al. [14] have studied the combination of a concentric annulus storage system with erythritol in combination with an absorption cooling system, while Sharma et al. [15] as well as Pawar et al. [16] have used the PCM for a solar cooking system. Kaizawa et al. [17] studied the technical feasibility for a waste heat transportation system with erythritol. Wang [18] has investigated a system with direct contact between a heat transfer fluid and PCM.

For the second sugar alcohol, xylitol, only a few publications concerning the utilization as PCM exist. Kaizawa et al. [7] have determined a decomposition temperature of 200 ${}^{\circ }C$, which is the limiting temperature for its use as thermal energy storage material. Seppälä et al. [19] have tested various additives to increase the speed of crystallization and the release of latent heat and have measured thermal properties. Methanol leads to a 33–170 times faster crystallization compared to the pure xylitol in a supercooled state at 22 ${}^{\circ }C$. Thermal properties of xylitol from various sources are listed in Table 1.

Investigation of the salt hydrate MgCl${}_{2}$·6H${}_{2}$O have been carried out by several authors. Lane [20] points out the incongruent melting behaviour of the salt hydrate and the occurrence of supercooling of 20 K. This phenomenon has appeared in the studies of Cantor [21] at DSC scale samples of only a few milligram, while experiments in test tubes with several grams of PCM have indicated virtually no supercooling. This volume dependency of the supercooling has been pointed out by Rathgeber et al. [12] as well who compared DSC results with T-history measurements. Neither supercooling nor phase separation were reported by Choi and Kim [22] and Gonçalves and Probert [23], but they have used a nucleating agent. Pilar et al. [24] have tested various nucleating agents in different compositions and reached a reduction of the supercooling from 37 K without additive to almost zero. The cycle stability of MCHH has been tested by El-Sebaii et al. [25] for PCM within unsealed and sealed containers [26]. In both studies, the extra water principle has been used to avoid the segregation problem during the phase change. In the fist study [25], the melting enthalpy decreased to only 45% of the start value after 500 cycles and the melting temperature shifted from 111.5 ${}^{\circ }C$ at the first cycle to 124 ${}^{\circ }C$ after 500 cycles. In the following studies, El-Sebaii et al. [26] have used sealed containers for the cycling experiments, which result in a decrease of the melting enthalpy of only 5% and a shift of the melting temperature of 5 K. Magnesiumchloride hexahydrate is found in different applications. Choi and Kim [22] have used the PCM for experiments with a finned and unfinned concentric tube arrangement while El-Sebaii et al. [25,26] have focused on solar cooking devices. Gonçalves and Probert [23] have applied MCHH, macro encapsulated in cans for a packed bed type storage system. Some important thermophysical properties of various authors are listed in Table 1.

Table 1 demonstrates some difficulties which are encountered when working with data from literature. The thermophysical properties vary from author to author, sometimes induced by the different measurement methods applied, but more often with the same equipment. The specimen itself is another parameter which is responsible for different results. The properties of a material may vary depending on its grade. Most manufacturers do not perform their own property measurements of technical grade material and rely on available data from analytical grade material [27]. This can cause large errors, when these values are set for the design of a thermal energy storage system. Due to economic aspects, only technical grade materials are investigated within this study, since the material selection aims on applicability in real scale storage systems. Furthermore, some properties are not available or at one specific temperature only, which complicates calculations or simulations, since missing values have to be assumed.

Thus, the aim of this work is to identify an appropriate material for energy storage applications between 90 and 150 ${}^{\circ }C$ and to evaluate its temperature dependent thermophysical properties. The results are transferred to linear equations which describe the experimental data within the standard deviation of the measurement and allow the utilization of the thermophysical properties for detailed simulations.

## 2. Material and Methods

### 2.1. Investigated PCM

The investigations were performed with PCM of technical grade purity. The purity was chosen due to economical reasons, since the investigations were conducted for a real scale storage application with a material demand of several tons. The supplier of the sugar alcohols erythritol and xylitol is Hamburg Fructose GmbH International, Hamburg, Germany with a price of 6.12 and 5.72 €/kg, respectively. The magnesiumchloride hexahydrate is from Schwarzmann GmbH, Laaber, Germany with a price of 0.77 €/kg.

### 2.2. Heat Capacity, Melting Temperature and Latent Heat

#### 2.2.1. DSC

Heat capacities, melting temperatures and the latent heat of the PCM samples were studied with a DSC 200 F3 Maia^®^ from Netzsch, Selb, Germany. The analysis was carried out in accordance with the procedure given by Gschwander et al. [33], but, due to the supercooling of the specimen, only the heating cycles were analysed. Three samples with masses of about 10 mg were taken from every PCM and placed as powder in 25 μL aluminium crucibles and sealed by cold welding. Each sample was exposed to three consecutive cooling and heating cycles with a heating and cooling rate of 0.5 K/min. The heating rate was determined with a heating rate test to ensure thermal equilibrium within the samples [33]. Since these small heating rates result in a bad signal-to-noise ratio within the sensible regions (pure solid and liquid state) of the PCM, additional measurements with heating rates of 10 K/min for determining the heat capacity were performed. All measurements were conducted under nitrogen atmosphere at 20 mL/min. Temperature and heatflow calibration were realised with pure water, gallium, indium, bismuth and zinc. A sapphire reference standard was applied for the determination of the heat capacities. The accuracy of the DSC for the enthalpy and heat capacity measurements can be assumed within a range of ±5% and ±3%, respectively [34]. The melting temperature $\vartheta_{m}$ was determined as the extrapolated onset temperature of the melting peak and the melting enthalpy $h_{m}$ was calculated with a linear baseline [35].

#### 2.2.2. Three-Layer-Calorimeter

The three-layer-calorimeter (3LC), manufactured by Laube [36], is an instrument to determine heat capacities, melting enthalpies and phase change temperatures of PCM. The advantage of the calorimeter is the comparatively high sample mass of about 100 g compared to commonly used sample masses of about 15 g in T-history methods and only a few mg in DSC instruments [37]. The bigger sample size allows investigations under conditions close to practical applications, like macro-encapsulation, and delivers realistic values for the volume dependent supercooling.

**Figure 1:**
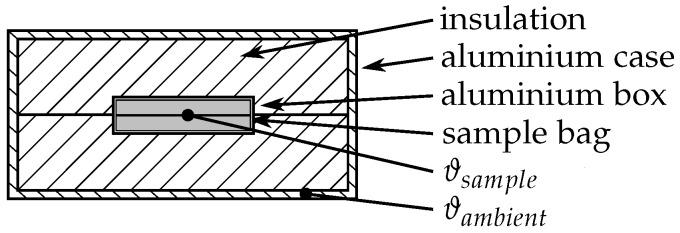
Sketch of the three-layer-calorimeter.

As shown in Figure 1, the 3LC consists of an aluminium box that is covered by an insulation placed inside an aluminium case. The PCM sample is welded in a foil bag made of FEP (fluorinated ethylene propylene) folded and placed inside the inner aluminium box. Thermocouples placed between the two halves of the folded sample bag and at the aluminium case measure the temperature of the sample and the ambient, respectively. The whole setup was placed in an oven and heated up to a temperature of 15 K below melting temperature of the investigated specimen. After reaching isothermal conditions (∼24 h), the oven temperature was increased to 15 K above the melting temperature. The temperature evolution of the sample $\vartheta_{sample}$ and the ambient $\vartheta_{ambient}$ within this temperature-step was recorded. For the examination of the solidification behaviour, the oven temperature was decreased to the initial temperature, 15 K below the melting temperature. A comparison with calibration data, deposited in the evaluation tool *WOTKA* delivered by the manufacturer of the 3LC, allows the determination of the phase change temperature, specific heat and phase change enthalpy of the sample.

### 2.3. Density

**Figure 2:**
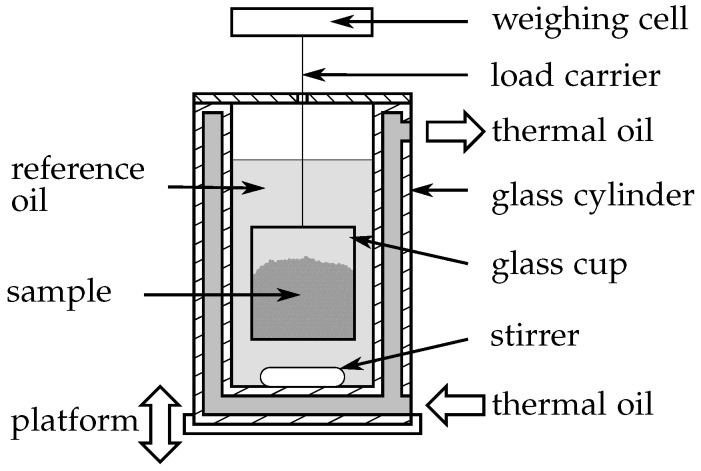
Sketch of the IMETER setup for density measurements.

Density measurements were performed with an IMETER, manufactured by Breitwieser [38]. The working principle is a hydrostatic weighing. As shown in Figure 2, a defined amount of the sample is filled as granulate in a quartz glass cup. The cup is connected to a weighing cell by a load carrier that consists of a thin tungsten wire. When the specimen is submerged into a liquid with known density, the volume of the sample can be obtained and thus the density of the specimen is predictable.

Since the investigated PCM xylitol, erythritol and MgCl${}_{2}$·6H${}_{2}$O are soluble in water, a reference oil (N100S from Paragon Scientific, Prenton, United Kingdom) with well known density was chosen instead. The reference oil and the glass cup are surrounded by a double-walled glass cylinder that is flushed by a thermal oil to adjust the sample temperature in a range between 20 ${}^{\circ }C$ up to 150 ${}^{\circ }C$. A magnetic stirrer ensures a uniform temperature within the measuring chamber. The densities are calculated by an equation proposed by Breitwieser [38]:

(1) $$\varrho_{sample}=\frac{\varrho_{reference}-\varrho_{ambient}}{1-\frac{W_{sample,reference}}{W_{ambient}}}+\varrho_{ambient}.$$

Therein, $\varrho_{reference}$ is the density of the reference liquid, $\varrho_{ambient}$ the density of the ambient air and $W_{sample,reference}$ and $W_{ambient}$ are the weighing values of the sample within the reference liquid and the ambient air. With the density values at different temperatures, the volumetric coefficient of thermal expansion $\alpha_{V}$ is calculated with:

(2) $$\alpha_{V}=\frac{\varrho_{0}-\varrho_{\vartheta }}{\varrho_{0}\left(\vartheta -\vartheta_{0}\right)}.$$

Therein, $\varrho_{0}$ is the density at a reference temperature (first measured temperature in the solid and liquid state), $\varrho_{\vartheta }$ the density at the temperature ϑ, and (ϑ − $\vartheta_{0}$) the temperature difference between the reference and the actual measurement. The change of density $\Delta \varrho_{sl}$ is defined as the difference between the solid ($\varrho_{s}$) and liquid density ($\varrho_{l}$) at the melting point with regard to the solid state:

(3) $$\Delta \varrho_{sl}=\frac{\varrho_{s}-\varrho_{l}}{\varrho_{s}}.$$

These densities are determined by extrapolating the results of both phases to the melting point. Due to the comparatively big specimen volume of about 12 mL, the sample can be assumed as representative. Therefore, only one sample was measured in detail at different temperatures. A second sample with only one density measurement at a defined temperature was then used to validate the results and to eliminate sample dependencies. For the measurement, the specimen was heated up to a defined temperature. After reaching a stationary state, five consecutive measuring points were recorded and the mean value and its uncertainty are calculated. The uncertainty of the results was calculated from the known uncertainties of the fluid density, the weighing cell and the temperature measurement with a coverage factor of *k* = 1.

### 2.4. Thermal Diffusivity and Conductivity

**Figure 3:**
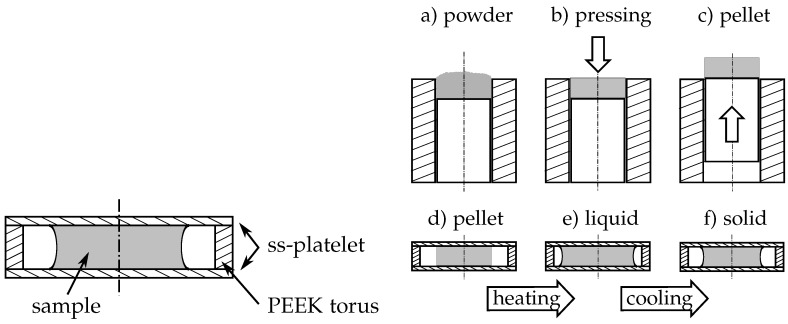
Sketch of the LFA sample holder (**left**) and steps of the sample preparation for the LFA measurements (**right**).

The thermal diffusivity of the investigated PCM is measured with a LFA (light flash apparatus) 447 NanoFlash^®^ from Netzsch, Selb, Germany. Three different samples of each PCM with five consecutive diffusivity measurements at different temperatures in the solid and the liquid phase were investigated. A specimen holder for low viscosity liquids was used for the measurements. As sketched in Figure 3, it consists of two stainless steel platelets that are separated by a PEEK (polyether ether ketone) torus. The torus has an inner diameter of 15 mm and the thickness of the torus and the platelets is 1.5 mm and 0.1 mm, respectively. The three parts are held together by a bolted housing not shown in the sketch. Normally, the liquid sample is filled in the specimen holder with a syringe through two small holes in the PEEK torus. Due to the high melting point of the investigated PCM (between 90 ${}^{\circ }C$ and 120 ${}^{\circ }C$), this procedure is not applicable. Instead of filling in as a liquid, the specimen was adapted in at solid state. Therefore, the PCM was milled to fine powder (Figure 3a) and afterwards pressed in a cylindrical mould (Figure 3b) with a diameter of 13.8 mm and a depth of 1.55 mm. The diameter difference between the pressed powder pellet and the PEEK torus allows for the expansion of the PCM during the phase change and the slightly greater thickness of the pellet ensures sufficient contact with the platelets. The pellet was then placed in the specimen holder (Figure 3d) and the whole assembly was inserted in the LFA. Each measurement started above the melting point of the specimen to ensure the thermal contact between the PCM and the platelets (Figure 3e). Afterwards, the sample was cooled down and the thermal diffusivity in the solid state was determined (Figure 3f). After each series of measurements, the solidified specimen was reviewed to make sure that there were no air bubbles within the sample and that there was contact of the PCM with the platelets. When the sample was fine, the thermal diffusivity had been calculated with the Proteus LFA Analysis software version 6.1.0 from Netzsch, Selb, Germany applying a three-layer-model (platelet—sample—platelet). According to Netzsch, the accuracy of the LFA measurement with this type of sample holder can be assumed within a range of ±5% (tested with water).

The thermal conductivity λ is calculated from the measured thermal diffusivity *a*, density ϱ and heat capacity $c_{p}$:

(4) $$\lambda =a\varrho c_{p}.$$

Since all of these quantities are afflicted by the uncertainty of the applied measurement devices, the combined uncertainty of the thermal conductivity is calculated using Gaussian propagation of uncertainty. The relative uncertainties of the devices specified by the manufacturer and applied for calculation are 5%, 3% and 0.1% for the thermal diffusivity, heat capacity and density measurement, respectively.

### 2.5. Cycling

Cycling experiments were performed to analyse the long time stability of the storage material. A PCM sample of about 10 mg was placed in an aluminium crucible and continuously heated up to 155 ${}^{\circ }C$ and cooled down to 55 ${}^{\circ }C$ in the DSC. The measurements were performed with sensitivity and temperature calibration for the applied heating rate of 10 K/min. The latent heat and temperature were determined after each cycle. Since it was not reasonable to measure and subtract the signal of the empty crucible for all cycles, the absolute value of the latent heat has been afflicted with errors. Therefore, the enthalpy of each cycle was related to the latent heat of the first cycle and thus the error of the empty pan was eliminated. The determination of the melting temperature was not affected by the empty crucible, but, due to comparability, the temperature after each cycle was related to the temperature of the first cycle as well.

## 3. Results and Discussion

### 3.1. Heat Capacity, Melting Temperature and Latent Heat

#### 3.1.1. DSC

**Figure 4:**
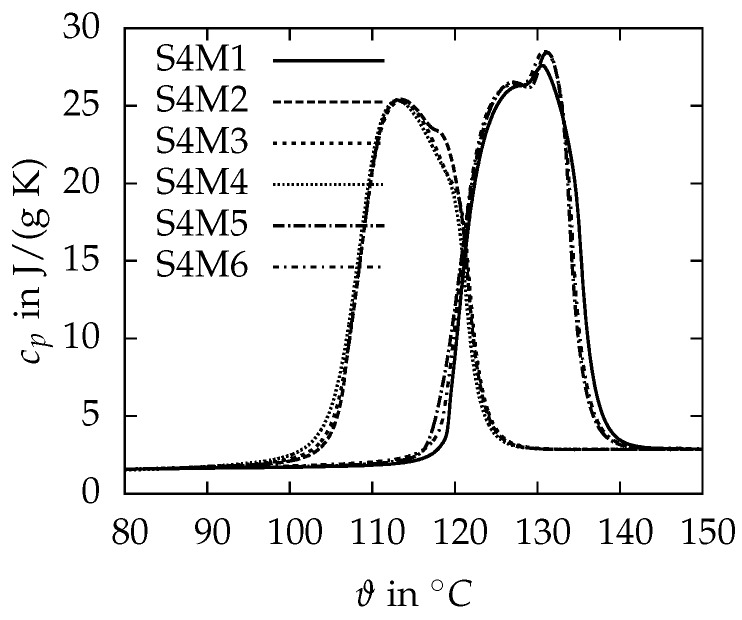
Heat capacity of erythritol at different melting cycles. The sample has two different melting points depending on the present crystal structure within the solid phase.

First, the measurements of the heat capacities $c_{p}$ performed with the DSC at high heating rates of 10 K/min are presented. The xylitol and erythritol samples show a strong supercooling behaviour. While for erythritol the crystallisation can be observed about 60 K below the melting point, the crystallisation for xylitol never starts after it is molten once. The onset of melting for xylitol is detected at 90 ± 1 ${}^{\circ }C$ and therefore lower than typical literature values of 92–94 ${}^{\circ }C$ [5,7,19,28,29,30] and the melting enthalpy of 237.6 ± 1.3 J/g, determined by the integration of the $c_{p}$ curve, agrees well with the result of 240.1 J/g published by Diarce et al. [29]. The heat capacity in the solid state at 20 ${}^{\circ }C$ is 1.27 ± 0.05 J/(g K), and, in the liquid state at 120 ${}^{\circ }C$, it is 2.73 ± 0.08 J/(g K). A study by Barone et al. [30] reports a heat capacity of 1.33 J/(g K) in the solid state and a lower value of 2.36 J/(g K) in the liquid state, but without detailed information about the corresponding temperature. Erythritol shows two different melting points. Figure 4 presents the heat capacity as a function of the temperature for six repeated measurements (M1–M6) of the same sample of erythritol (S4). While measurements M2–M4 have an onset of melting of 105.1 ± 0.1 ${}^{\circ }C$, the melting starts at 118.1 ± 0.6 ${}^{\circ }C$ for M1, M5 and M6. The reason for the two melting points is the occurrence of different crystal structures within the solid phase as reported by Lopes Jesus et al. [8]. The higher melting temperature fits well with 118 and 118.4 ${}^{\circ }C$ from Talja and Roos [28] and Sari et al. [10]. The melting enthalpy determined by integration of the $c_{p}$ curve is 352.9 ± 0.7 J/g and 316 ± 1 J/g for the 118 ${}^{\circ }C$ and 105 ${}^{\circ }C$ melting peak, respectively. These values meet the broad range of available results between 315–379.57 J/g from other studies [5,7,9,10,12,28,29]. The heat capacity in the solid state at 20 ${}^{\circ }C$ is 1.34 ± 0.09 J/(g K), and, in the liquid state at 150 ${}^{\circ }C$, it is 2.87 ± 0.03 J/(g K), which is in good agreement with 1.38 J/(g K) and 2.76 J/(g K) from the studies of Kakiuchi et al. [31]. The strong supercooling and the occurrence of different melting ranges restrict the utilisation of the sugar alcohols as PCM. Therefore, no further detailed DSC investigations were carried out with xylitol and erythritol.

**Figure 5:**
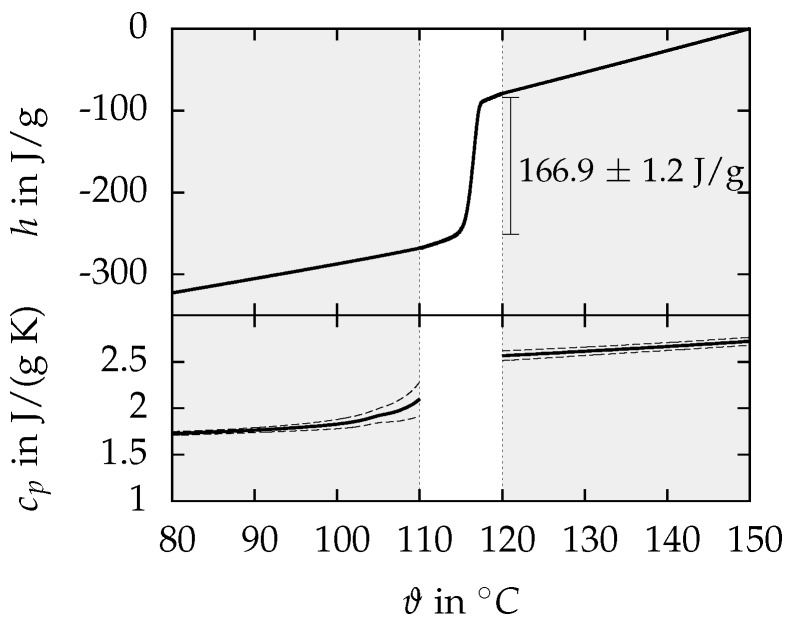
Enthalpy *h* and heat capacity $c_{p}$ as a function of the temperature ϑ for MCHH. The upper graph presents the mean value of three samples. The grey areas are determined with the c${}_{p}$-comparative method and the melting peak with a sensitivity calibration. The dashed lines represent the standard deviation.

The MgCl${}_{2}$·6H${}_{2}$O samples show a smaller supercooling of maximum 30 K below the melting point. In the upper part of Figure 5, the measured enthalpy *h* is visualized as a function of the temperature ϑ for melting. The upper graph presents the mean value of three different samples with three consecutive melting cycles per sample. As explained in Section 2.2, different measuring methods and heating rates were applied for the regions of sensible and latent heat. The areas highlighted in grey were determined with the c${}_{p}$-comparative method and a heating rate of 10 K/min, while the melting peak was determined with a heating rate of 0.5 K/min and a sensitivity calibration. The mean value of the latent heat is 166.9 ± 1.2 J/g between 114 and 118 ${}^{\circ }C$, and the onset and offset temperatures of melting are 115.1 ± 0.1 ${}^{\circ }C$ and 117.4 ± 0.1 ${}^{\circ }C$, respectively. The latent heat agrees well with 167 J/g from the results of Cantor [21] and 169 J/g from the studies of Rathgeber et al. [12].

The mean value of the heat capacity as a function of the temperature is plotted in the lower part of Figure 5. Within the solid region, between 80 and 110 ${}^{\circ }C$, $c_{p}$ rises progressively with increasing temperature while in the liquid region, between 120 and 150 ${}^{\circ }C$, $c_{p}$ increases linearly. The dashed lines represent the standard deviation of the three investigated samples with three measuring cycles at each sample. The heat capacity in the solid state at 100 ${}^{\circ }C$ is 1.83 ± 0.06 J/(g K), and, in the liquid state at 120 ${}^{\circ }C$, it is 2.57 ± 0.06 J/(g K). The solid state heat capacity is significantly smaller compared to 2.25 J/(g K) reported by Lane [20], but the value in the liquid state of 2.61 J/(g K) agrees well with our measurements. The heat capacity as a function of temperature in the liquid state can be described with a linear equation (Table 2) with a maximum deviation of 0.2% to the measured values. In the solid state, there seem to occur first phase transition effects above 100 ${}^{\circ }C$ in some of the samples, which cause a deviation from the linear behaviour between 80 and 100 ${}^{\circ }C$. Nevertheless, it is possible to describe the whole temperature range between 80 and 110 ${}^{\circ }C$ with one linear equation within the standard deviation of the measurements. When applying the parameters listed in Table 2, the maximum deviation between calculated and measured values is −8.3% at 110 ${}^{\circ }C$ compared to a standard deviation of the measurements of ±9%. Some key results of the measurements are summarized in Table 4.

#### 3.1.2. Three-Layer-Calorimeter

Despite the big sample size of about 100 g, it is not possible to solidify xylitol after it was molten once, and, therefore, no results can be evaluated. Erythritol starts to melt at 118.2 ${}^{\circ }C$, which corresponds with the onset of melting examined in some melting cycles of the DSC investigations. The crystallisation of the liquid erythritol starts at 71.2 ${}^{\circ }C$, which results in a supercooling of 47 K. This is about 13 K lower compared to the DSC results but is nevertheless too large for the desired application. The melting enthalpy is 337 J/g between 110 and 125 ${}^{\circ }C$ and therefore 5% lower than the DSC results.

**Figure 6:**
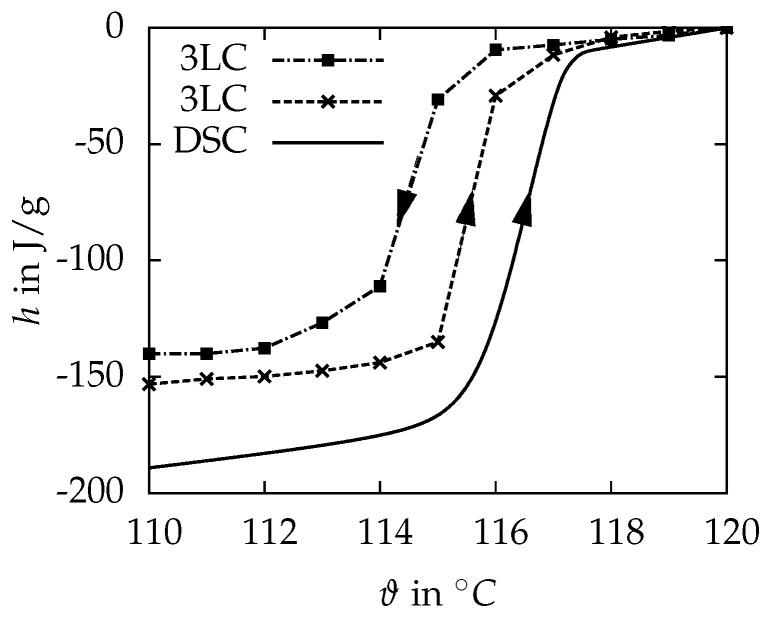
Enthalpy *h* as a function of the temperature ϑ for MCHH. The solid line is the result of the DSC measurement (Figure 5) and the dashed lines connect the resulting measuring points from the 3LC. The arrows indicate the direction of temperature change within the measurements.

The MCHH starts to melt at 115.8 ${}^{\circ }C$, which agrees well with the onset of melting examined within the DSC measurements. The crystallisation of the liquid MCHH starts at 113 ${}^{\circ }C$, which results in a supercooling of 2.8 K. It is about 27 K lower compared to the DSC results. Figure 6 compares the results from the DSC measurement with the results from the three-layer-calorimeter. The phase change enthalpy for the melting cycle is 143.4 J/g between 114 and 118 ${}^{\circ }C$ and therefore 14% lower than the DSC result. A reason for this large deviation has not been identified yet. Figure 6 presents the solidification cycle of the MCHH as well. Some key results of the measurements are summarized in Table 4.

### 3.2. Density

**Figure 7:**
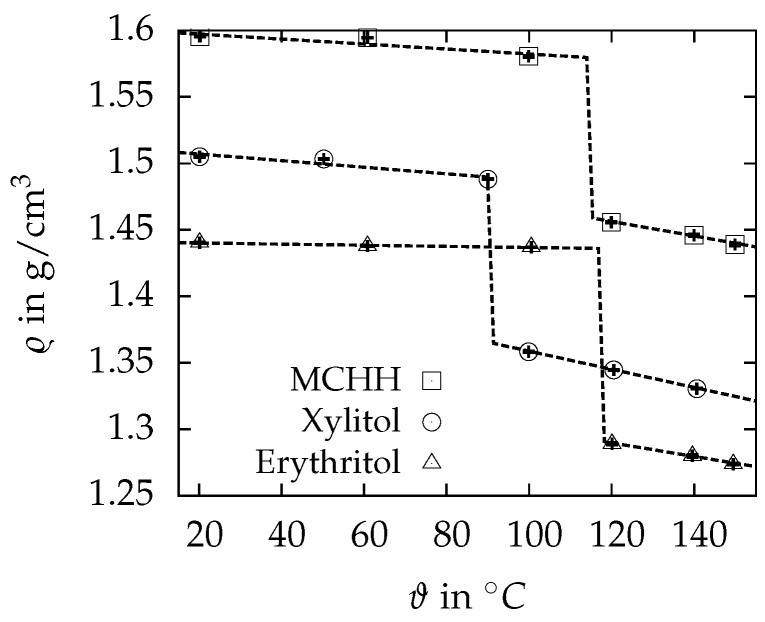
Density ϱ as a function of the temperature ϑ of the investigated PCM. The dashed line represents the linear fit of the measured points.

The results of the density measurements are summarized in Figure 7. The error bars are the measurement uncertainties, and they are smaller than 0.1% for all measured points. The standard deviation of the five measurements at each temperature lies within the range of the uncertainties. The volumetric coefficients of thermal expansion $\alpha_{V}$ are calculated with Equation (2) and the change of density $\Delta \varrho_{sl}$ with Equation (3).

Magnesiumchloride hexahydrate has the highest density of 1.5955 ± 0.0002 $g/{\mathrm{cm}}^{3}$ at 20 ${}^{\circ }C$, followed by xylitol and erythritol with 1.5050 ± 0.0004 $g/{\mathrm{cm}}^{3}$ and 1.4404 ± 0.0005 $g/{\mathrm{cm}}^{3}$, respectively. The results are in good agreement within 3% with the values stated by Mehling and Cabeza [5]. Within the solid region, xylitol has the highest volumetric coefficient of thermal expansion with 1.64 · 10^−4^/K, followed by MCHH with 1.17 · 10^−4^/K and erythritol with 2.94 · 10^−5^/K. In the liquid state, the order is xylitol, erythritol and MCHH with values of 5.02 · 10^−4^/K, 3.95 · 10^−4^/K and 3.76 · 10^−4^/K, respectively. For the calculation of $\alpha_{V}$, a linear behaviour of the change of density was assumed. This assumption results in a maximum deviation of 0.3% between measured values and the linear fit. The density change from solid to liquid is 10.1% for erythritol, followed by 8.4% for xylitol and 7.7% for MCHH. For these values, the measured results were extrapolated to the melting point of the specimen. Some key results of the measurements are summarized in Table 4.

### 3.3. Thermal Diffusivity and Conductivity

**Figure 8:**
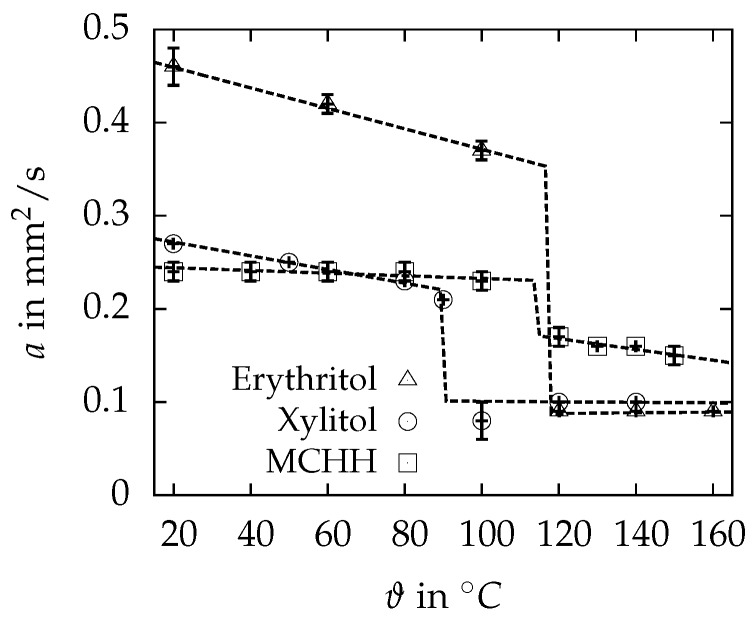
Thermal diffusivity *a* of the investigated PCM. The error bars are the standard deviation from three different samples and five shots at each temperature. The dashed line represents the linear fit of the points, used to calculate the temperature dependency of *a*, and extrapolated to the melting point of the PCM.

The results of the thermal diffusivity measurements are summarized in Figure 8. The error bars are the standard deviation calculated from three different samples and five shots at each temperature. The measurements with MCHH and erytritol started at the high temperatures in the liquid state. Xylitol started at the solid state, since, due to the high supercooling, no solidification was possible after the sample had been molten once.

The thermal diffusivity of all specimens is higher in the solid than in the liquid state. Furthermore, it decreases with increasing temperatures in the solid and is nearly temperature independent within the liquid. Only MCHH indicates a remarkable decrease of the thermal diffusivity in the liquid state with increasing temperatures. The reproducibility is good when measuring the liquid, pointed out by the standard deviation of less than 4% for all specimens. Within the solid region, higher deviation can be observed, possibly caused by a bad or partially lost contact between the samples and the platelets of the sample holder. Nevertheless, the standard deviation is less than 5% for all measured points in the solid state. A significantly higher standard deviation of about 27% can only be noticed for the xylitol sample at 100 ${}^{\circ }C$, which could be caused by a not completely molten sample.

Erythritol has the highest thermal diffusivity of 0.456 ± 0.018 $mm^{2}/s$ at 20 ${}^{\circ }C$, followed by xylitol and MCHH with 0.270 ± 0.002 $mm^{2}/s$ and 0.244 ± 0.011 $mm^{2}/s$, respectively. In the liquid state at 120 ${}^{\circ }C$, both sugar alcohols have nearly the same thermal diffusivities with 0.088 ± 0.001 $mm^{2}/s$ and 0.100 ± 0.001 $mm^{2}/s$ for erythritol and xylitol, while MCHH has the highest value of 0.173 ± 0.009 $mm^{2}/s$.

As indicated in Figure 8, the mean values of the measured thermal diffusivity can be well described with a linear fit. The obviously deviating point for xylitol at 100 ${}^{\circ }C$ was left out for this calculation. The linear equations describing the temperature dependent behaviour of each PCM in the solid and liquid state are summarized in Table 3. The maximum relative deviations between measured thermal diffusivities and the linear approximations are 1.3%, 2.6% and 4.7% for erythritol, xylitol and MCHH, respectively.

Since there are no data for the thermal diffusivities available in the literature, it is not possible to compare the measured results. Another option is the comparison of the thermal conductivity, calculated from the measured thermal diffusivity, density and heat capacity. The combined uncertainties of the determined conductivities are calculated with a coverage factor of *k* = 1.

MCHH has a thermal conductivity of 0.70 ± 0.05 W/(m K) in the solid state at 110 ${}^{\circ }C$ and 0.63 ± 0.04 W/(m K) in the liquid state at 120 ${}^{\circ }C$. The solid state thermal conductivity is in good agreement with the value of 0.704 W/(m K) from Mehling and Cabeza [5] and Lane [20], and the liquid state thermal conductivity from this work is 10% higher compared to the literature value of 0.570 W/(m K). The thermal conductivities of erythritol are 0.89 ± 0.06 W/(m K) and 0.33 ± 0.02 W/(m K) for the solid state at 20 ${}^{\circ }C$ and the liquid state at 140 ${}^{\circ }C$, respectively. The thermal conductivity in the liquid state agrees with the value of Mehling and Cabeza [5], but, within the solid state, it is 20% higher and beyond the range of the combined uncertainty. Xylitol has a thermal conductivity of 0.52 ± 0.04 W/(m K) in the solid state at 20 ${}^{\circ }C$ and 0.36 ± 0.03 W/(m K) in the liquid state at 140 ${}^{\circ }C$, and there are no comparative figures in the literature. Some key results of the measurements are summarized in Table 4.

### 3.4. Cycling

**Figure 9:**
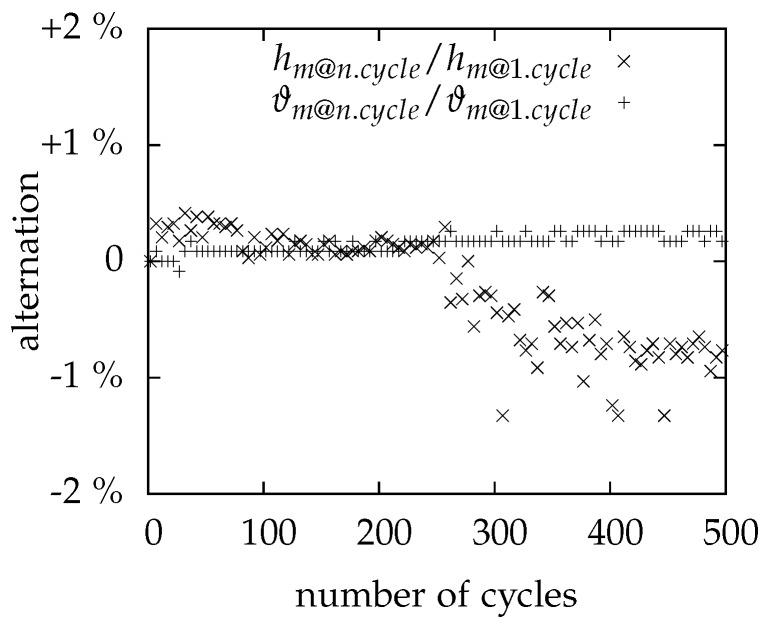
Results of the cycling test with MCHH. The enthalpies and temperatures of each cycle are related to the value of the first cycle.

Figure 9 demonstrates the results of the cycling experiments performed with MCHH. The melting enthalpies ($h_{m@n.cycle}/h_{m@1.cycle}$) and temperatures ($\vartheta_{m@n.cycle}/\vartheta_{m@1.cycle}$) of each cycle are related to the value of the first cycle. The results indicate a small shift of the melting temperature up to higher values, but the change is less than 0.3 K in absolute values. The melting enthalpy increases within the first cycles and decreases afterwards subsequently to about 99% of the starting value. There seems to be a plateau at that level, but further investigations are necessary for verification, especially with samples at application scale. El-Sebaii et al. [25,26] have reported stronger fluctuations of the melting temperature and the melting enthalpy, but they used larger samples and hence segregation effects may appear stronger. There are no results for erythritol and xylitol since their strong and irregular supercooling requires a lot of additional effort for the experiments.

## 4. Conclusions

The thermophysical properties of the sugar alcohols xylitol and erytrhitol and of the salt hydrate magnesium chloride hexahydrate were investigated with different measuring techniques. The melting and crystallization temperatures as well as the enthalpies and heat capacities were determined with a DSC and a three-layer-calorimeter, which allows the examination of bigger sample sizes. The thermal diffusivity was measured with the LFA measuring technique and the densities of the PCM were determined with a hydrostatic weighing.

Table 4 summarizes the results of the three investigated PCMs. The two sugar alcohols erythritol and xylitol show considerable phase change enthalpies. In combination with the high densities, remarkable volumetric storage densities of up to 450 $MJ/m^{3}$ are obtainable. The thermal diffusivity is comparatively high, especially for erythritol, with the highest values of the three candidates within the solid region. A high thermal diffusivity and consequently a high thermal conductivity is important for the PCM storage performance, especially at the solidification process, since the heat transfer is dominated by the continuously growing solid layer. Nevertheless, both sugar alcohols are not suitable for mobile thermal energy storage applications in the present form. The occurrence of different melting points for erythritol and the high supercooling of both candidates as well as their prices are great drawbacks. Therefore, further investigations to understand and reduce these negative effects are necessary to make theses attractive sugar alcohols applicable for technical applications.

The studied salt hydrate MgCl${}_{2}$·6H${}_{2}$O has a considerable supercooling at DSC scale samples, but an acceptable value of only 2.8 K at samples sizes of 100 g, which is a typical size for storage applications with macro-encapsulation. The melting enthalpy is 166.9 J/g, therefore about half of erythritol, and the achievable volumetric storage density is only 240 $MJ/m^{3}$. Despite these comparatively low storage densities, MCHH is the favourable candidate as PCM for waste heat transportation systems within this study. Linear equations describe the measured properties and allow the easy access to detailed material parameters for e.g. simulations.

## Figures and Tables

**Table 1:** Summary of literature values for the investigated PCMs.

Property	Erythritol	Source	Xylitol	Source	MCHH	Source
$\vartheta_{m}$ in ${}^{\circ }C$	117–120	[5,7,9,10,28,29]	92–94	[5,7,19,28,29,30]	110.8–117.5	[5,20,21,24,25,26]
$h_{m}$ in J/g	315–379.57	[5,7,9,10,12,28,29]	232–280	[5,7,19,28,29,30]	133.9–200	[5,12,20,21,24,25,26]
$c_{p,s}$ in J/(g K)	1.38 (20 ${}^{\circ }C$)	[31]	1.33	[30]	2.25 (100 ${}^{\circ }C$)	[20]
					2.1 (25 ${}^{\circ }C$)	[32]
$c_{p,l}$ in J/(g K)	2.76 (140 ${}^{\circ }C$)	[31]	2.36	[30]	2.61 (120 ${}^{\circ }C$)	[20]
$\lambda_{s}$ in W/(mK)	0.733 (20 ${}^{\circ }C$)	[5]	-	-	0.704 (110 ${}^{\circ }C$)	[5,20]
$\lambda_{l}$ in W/(mK)	0.326 (140 ${}^{\circ }C$)	[5]	-	-	0.570 (120 ${}^{\circ }C$)	[5,20]
$\varrho_{s}$ in $g/{\mathrm{cm}}^{3}$	1.480 (20 ${}^{\circ }C$)	[5]	1.500 (20 ${}^{\circ }C$)	[5]	1.569 (20 ${}^{\circ }C$)	[5,20]
$\varrho_{l}$ in $g/{\mathrm{cm}}^{3}$	1.300 (140 ${}^{\circ }C$)	[5]	-	-	1.450 (120 ${}^{\circ }C$)	[5,20]
					1.422 (128 ${}^{\circ }C$)	[32]

**Table 2:** Factors for the linear equation $c_{p}\left(\vartheta \right)=d_{1}$·$\vartheta +d_{2}$ to describe the temperature dependency of the heat capacity of MCHH.

Range	$d_{1}$	$d_{2}$
solid (80–110 ${}^{\circ }C$)	$7.00\times 10^{-3}$	1.155
liquid (120–150 ${}^{\circ }C$)	$5.145\times 10^{-3}$	1.945

**Table 3:** Factors for the linear equation $a\left(\vartheta \right)=e_{1}$·$\vartheta +e_{2}$ to describe the temperature dependency of the thermal diffusivity.

Range	$e_{1}$	$e_{2}$
Erythritol		
s. (20–118 ${}^{\circ }C$)	$-1.099\times 10^{-3}$	$4.813\times 10^{-1}$
l. (118–150 ${}^{\circ }C$)	$3.338\times 10^{-5}$	$8.390\times 10^{-2}$
Xylitol		
s. (20–90 ${}^{\circ }C$)	$-7.351\times 10^{-4}$	$2.865\times 10^{-1}$
l. (90–140 ${}^{\circ }C$)	$-2.666\times 10^{-5}$	$1.035\times 10^{-1}$
MgCl${}_{2}$·6H${}_{2}$O		
s. (20–115 ${}^{\circ }C$)	$-1.437\times 10^{-4}$	$2.471\times 10^{-1}$
l. (115–150 ${}^{\circ }C$)	$-5.794\times 10^{-4}$	$2.377\times 10^{-1}$

**Table 4:** Results of the three investigated PCMs including the standard deviation of the measurements. The results for the thermal conductivity are calculated from the measured thermal diffusivities *a*, densities ϱ and heat capacities $c_{p}$ and the deviation is the combined uncertainty of the applied measurement devices.

Property	Erythritol	Xylitol	MCHH
$\vartheta_{m,DSC}$ in ${}^{\circ }C$	105.1 ± 0.1	90 ± 1	115.1 ± 0.1
	118.1 ± 0.6		
$\vartheta_{m,3LC}$ in ${}^{\circ }C$	118.2	-	115.8
$\Delta \vartheta_{sup,DSC}$ in K	60	>90	30
$\Delta \vartheta_{sup,3LC}$ in K	47	-	2.8
$h_{m,DSC}$ in J/g	316 ± 1 (90–135 ${}^{\circ }C$)	237.6 ± 1.3 (70–116 ${}^{\circ }C$)	166.9 ± 1.2 (114–118 ${}^{\circ }C$)
	352.9 ± 0.7 (110–145 ${}^{\circ }C$)		
$h_{m,3LC}$ in J/g	337 (110–125 ${}^{\circ }C$)	-	143.4 (114–118 ${}^{\circ }C$)
$c_{p,s}$ in J/(gK)	1.34 ± 0.09 (20 ${}^{\circ }C$)	1.27 ± 0.05 (20 ${}^{\circ }C$)	1.83 ± 0.06 (100 ${}^{\circ }C$)
$c_{p,l}$ in J/(gK)	2.87 ± 0.03 (150 ${}^{\circ }C$)	2.73 ± 0.08 (120 ${}^{\circ }C$)	2.57 ± 0.06 (120 ${}^{\circ }C$)
$a_{s,20\;{}^{\circ }C}$ in $mm^{2}/s$	0.456 ± 0.018	0.270 ± 0.002	0.244 ± 0.011
$a_{l,120\;{}^{\circ }C}$ in $mm^{2}/s$	0.088 ± 0.001	0.100 ± 0.001	0.173 ± 0.008
$\varrho_{s,20\;{}^{\circ }C}$ in $g/{\mathrm{cm}}^{3}$	1.4404 ± 0.0005	1.5050 ± 0.0004	1.5955 ± 0.0002
$\alpha_{V,s(20\;{}^{\circ }C\cdots \vartheta_{m})}$ in 1/K	2.94 · 10^−5^	1.64 · 10^−4^	1.17 · 10^−4^
$\varrho_{l,120\;{}^{\circ }C}$ in $g/{\mathrm{cm}}^{3}$	1.2891 ± 0.0008	1.3446 ± 0.0003	1.4557 ± 0.0004
$\alpha_{V,l(\vartheta_{m}\cdots 150\;{}^{\circ }C)}$ in 1/K	3.95 · 10^−4^	5.02 · 10^−4^	3.76 · 10^−4^
$\Delta \varrho_{sl}$ in %	10.1	8.4	7.7
$\lambda_{s}$ in W/(m K)	0.89 ± 0.06 (20 ${}^{\circ }C$)	0.52 ± 0.04 (20 ${}^{\circ }C$)	0.70 ± 0.05 (110 ${}^{\circ }C$)
$\lambda_{l}$ in W/(m K)	0.33 ± 0.02 (140 ${}^{\circ }C$)	0.36 ± 0.03 (140 ${}^{\circ }C$)	0.63 ± 0.04 (120 ${}^{\circ }C$)

## Abbreviations

The following abbreviations are used in this manuscript:

3LC		3-layer-calorimeter
DSC		Differential scanning calorimeter
FEP		Fluorinated ethylene propylene
LFA		Light flash apparatus
LHTES		Latent heat thermal energy storage
M		Measurement
MCHH		Magnesiumchloride hexahydrate
PEEK		Polyether ether ketone
PCM		Phase change material
ss		Stainless steel
S		Sample
*a*	$mm^{2}/s$	Thermal diffusivity
$c_{p}$	J/(g K)	Heat capacity
$d_{1}$, $d_{2}$		Factors for linear equations
$e_{1}$, $e_{2}$		Factors for linear equations
*h*	J/g	Enthalpy
*k*		Coverage factor
*W*	g	Weighing value
$\alpha_{V}$	1/K	Volumetric coefficient of thermal expansion
$\Delta \varrho_{sl}$	%	Change of density from solid to liquid state
ϑ	${}^{\circ }C$	Temperature
$\Delta \vartheta_{sup}$	K	Supercooling
λ	W/(mK)	Thermal conductivity
ϱ	$g/{\mathrm{cm}}^{3}$	Density
${□}_{0}$		Reference state
${□}_{l}$		Liquid (state)
${□}_{m}$		Melting
${□}_{s}$		Solid (state)
